# Telocytes subtypes in human urinary bladder

**DOI:** 10.1111/jcmm.12375

**Published:** 2014-08-19

**Authors:** Maria-Giuliana Vannucchi, Chiara Traini, Daniele Guasti, Del Popolo Giulio, Maria-Simonetta Faussone-Pellegrini

**Affiliations:** a Department of Experimental and Clinical Medicine, Histology and Embryology Research Unit, University of FlorenceFlorence, Italy; b Dept of Neuro-Urology, Careggi University HospitalFlorence, Italy

**Keywords:** lamina propria, sub-urothelium and submucosa, detrusor, mast cells, myofibroblasts, CD34, PDGFRα, c-Kit, αSMA, calreticulin

## Abstract

Urinary bladder voiding is a complex mechanism depending upon interplay among detrusor, urothelium, sensory and motor neurons and connective tissue cells. The identity of some of the latter cells is still controversial. We presently attempted to clarify their phenotype(s) in the human urinary bladder by transmission electron microscopy (TEM) and immunohistochemistry. At this latter aim, we used CD34, PDGFRα, αSMA, c-Kit and calreticulin antibodies. Both, TEM and immunohistochemistry, showed cells that, sharing several telocyte (TC) characteristics, we identified as TC; these cells, however, differed from each other in some ultrastructural features and immunolabelling according to their location. PDGFRα/calret-positive, CD34/c-Kit-negative TC were located in the sub-urothelium and distinct in two subtypes whether, similarly to myofibroblasts, they were αSMA-positive and had attachment plaques. The sub-urothelial TC formed a mixed network with myofibroblasts and were close to numerous nerve endings, many of which nNOS-positive. A third TC subtype, PDGFRα/αSMA/c-Kit-negative, CD34/calret-positive, ultrastructurally typical, was located in the submucosa and detrusor. Briefly, in the human bladder, we found three TC subtypes. Each subtype likely forms a network building a 3-D scaffold able to follow the bladder wall distension and relaxation and avoiding anomalous wall deformation. The TC located in the sub-urothelium, a region considered a sort of sensory system for the micturition reflex, as forming a network with myofibroblasts, possessing specialized junctions with extracellular matrix and being close to nerve endings, might have a role in bladder reflexes. In conclusions, the urinary bladder contains peculiar TC able to adapt their morphology to the organ activity.

## Introduction

Urinary bladder voiding is a complex mechanism depending upon interplay between detrusor smooth muscle cells, urothelium, sensory and motor neurons 1,2. In the recent years, attention has been focused also on the connective tissue cells located either in the *lamina propria*, particularly those underlying the urothelium 3–7, or close to or intermingled with the detrusor muscle bundles 7,8.

The *lamina propria* of the human urinary bladder consists of a connective tissue that is loose in its upper part, the sub-urothelial layer and dense in the deeper portion, the submucosa, up to the detrusor. The sub-urothelial layer is thinner than the submucosa and richer in both resident and migrated cells, nerves and blood vessels. The identity of some of the resident cells is still controversial. Indeed, close to the well-known fibroblasts 9,10 and mast cells, the authors refer of cells variably named although having some similar ultrastructural and/or immunohistochemical features. These cells are named: interstitial cells (IC) 3,6,11, interstitial cells of Cajal (ICC) 2,12–19, interstitial cell-like Cajal (ICLC) 3,5,8,20, telocytes (TC) 4,21, fibroblast-like cells (FLC) 8. Somebody among these cells encloses the myofibroblasts 9,10,22,23 notwithstanding these cells can be easily recognized because of their αSMA-positivity and typical ultrastructural features 24.

In summary, the main ambiguity is among IC, ICC, ILC, TC and FLC and it stems from the diverse methodologies utilized, immunohistochemistry *versus* transmission electron microscopy (TEM), and from the lack of a reliable marker for the identification of each cell type. Therefore, the necessity to ascertain whether all those names correspond to one or more types of cells is urgent.

The term IC is quite vague but still used 11. The definition of ICC, the pacemaker cells of the gut 25, comes from the observation of c-Kit-positive cells in the guinea pig and human bladder wall 12–20. As other researchers were unable to find c-Kit-positive cells in the mouse 7, it is still matter of debate whether canonical ICC are really present in the urinary bladder wall. With regard to the term ICLC, there is a general agreement to substitute it with the more precise term TC recently introduced 26–31. Up-to-date TC is a cell type identifiable with certainty under the TEM 26–32. Immunohistochemistry, performed in a variety of organs, shows an enormous phenotypical heterogeneity for the TC, but few markers have been seen to label most of them. Among these markers, the most reliable seems to be the CD34 26,27. Recent immunohistochemical studies on human bladder showed CD34-positive cells (called ICLC) in the detrusor 8 and in the submucosa 5, while cells (formerly called ‘TC’ and recently ‘fibroblasts with myoid features’) in the upper *lamina propria* were CD34-negative but αSMA-positive 3–5. Besides, these authors also reported that these cells were also PDGFRα-positive and c-Kit-negative 5. These findings allow supposing that, at least in the human bladder, there are two distinct types of TC. Cells formerly called FLC have been recently seen to be PDGFRα-positive but c-Kit-negative in mouse and rat urinary bladder; in the absence of their identification with a specific IC type, these cells are presently called PDGFRα-positive cells 7,33. PDGFRα/CD34-positive, c-Kit-negative cells have been found in the entire wall of human gastrointestinal tract 34,35 and identified as TC 34.

Briefly, we feel to can reasonable reduce the previous nomenclature for the resident cells present in the connective tissue of the bladder wall, but how many types these cells are and their exact identification and characterization according to the various bladder layers (sub-urothelium, submucosa, detrusor), are still undetermined. Taking into account the importance that the connective tissue cells have in regulating the bladder function and the possibility that alterations in their phenotype be implicated in neurological and not neurological bladder dysfunction, we consider useful the attempt to clarify their exact phenotype(s) by combining TEM and immunohistochemical observations. At the latter aim, we used CD34, PDGFRα, αSMA, c-Kit and calreticulin antibodies.

## Materials and methods

### Sample collection

Samples from the lateral wall of human bladders were retrieved on course of cystectomia by four patients (mean age: 75 ± 3 years) operated for cancer. All the patients signed an informed consent form, and the study complied with the principles of the Declaration of Helsinki and was approved by the local Institutional Review Board. Immediately after surgery, two full-thickness specimens were obtained from each sample far from the lesion and processed for immunohistochemistry and electron microscope studies, respectively. Sections from each sample were H/E stained to verify infiltrates or other pathological signs were not present.

### Immunohistochemistry

Full-thickness specimens were fixed in 4% paraformaldehyde in 0.1 M PBS (pH 7.4) over night (ON) at 4°C, dehydrated in a graded ethanol series, cleared in xylene and embedded in paraffin. Then the sections (5 μm thick) were cut by using a rotary microtome (MR2, Boeckeler Instruments Inc., Tucson, AZ, USA). The sections were deparaffinized as usual and for antigen retrieval boiled for 10 min. in sodium citrate buffer (10 mM, pH 6.0, Bio-Optica, Milan, Italy) or treated for 20 min. at 90–92°C in Tris buffer (10 mM) with EDTA (1 mM, pH 9.0), as appropriate. After antigen retrieval phase, the sections were washed in PBS, incubated in 2 mg/ml glycine (AppliChem, Darmstadt, Germany) for 10 min., to quench autofluorescence caused by the elastic fibres and blocked for 20 min. at room temperature (RT) with 1% BSA in PBS. The primary antibodies diluted in PBS were applied ON at 4°C. Information on primary antibody sources and used concentrations is shown in Table1. The day after, the slides were washed in PBS and incubated for 2 hrs at RT in the dark with appropriate fluorochrome-conjugated (Alexa Fluor 488-or 568-conjugated) secondary antibodies (goat anti-rabbit, goat antimouse or donkey anti-goat; Invitrogen, San Diego, CA, USA) diluted 1:333 in PBS. Tissue sections were then thoroughly washed in PBS and mounted in an aqueous medium (Sigma-Aldrich, St. Louis, MO, USA). Double labelling was performed as follows: after the first incubation as described above, the sections were incubated again with another primary antibody and with the specific secondary antibody. Negative controls were simultaneously performed omitting the primary antibody, to exclude the presence of non-specific immunofluorescence staining. The immunoreaction products were observed under an epifluorescence Zeiss Axioskop microscope (Mannheim, Germany), by using 488-and 568-nm excitation wavelength for the green and red fluorescent labels and the fluorescence images were captured by using a Leica DFC310 FX 1.4-megapixel digital camera, equipped with the Leica software application suite LAS V3.8 (Leica Microsystems, Milan, Italy).

**Table 1:** Details of primary antibodies

Primary antibody	Host	Antigen retrieval	Working dilution IHC	Source
PDGFRα	Goat	Sodium citrate buffer, pH 6.0 Tris-EDTA buffer, pH 9.0	1:100	Catalogue no. AF-307-NA; R&D Systems, Minneapolis, MN, USA
CD34	Mouse	Sodium citrate buffer, pH 6.0 Tris-EDTA buffer, pH 9.0	1:50	Catalogue no. M7165; Dako, Glostrup, Denmark
c-Kit	Rabbit	Tris-EDTA buffer, pH 9.0	1:300	Catalogue no. A4502; Dako
αSMA	Mouse	Tris-EDTA buffer, pH 9.0	1:500	Catalogue no. A-2547; Sigma-Aldrich, St. Louis, MO, USA
PGP9.5	Rabbit	Sodium citrate buffer, pH 6.0	1:200	Catalogue no. AB1761; Chemicon-Millipore, Temecula, CA, USA
Calreticulin	Chicken	Tris-EDTA buffer, pH 9.0	1:200	Catalogue no. PA1-902A; Thermo Scientific, Runcorn, UK
nNOS	Rabbit	Sodium citrate buffer, pH 6.0 Tris-EDTA buffer, pH 9.0	1:1500	Catalogue no. AB5380; Chemicon-Millipore

### Electron microscopy

Specimens of urothelium plus *lamina propria* and specimens of detrusor plus *lamina propria* were fixed in Karnowsky (paraformaldehyde 8% in distilled water and 0.2 M PBS containing 0.055 g/l NaPO_4_ and 0.04 M Lysine, added with 0.5% glutaraldehyde) ON at 4°C, and then post-fixed with 1% osmium tetroxide in 0.1 M PBS for 2 hrs at 4°C, dehydrated in graded series of acetone and embedded in Epon by using flat moulds. Semi-thin sections were obtained with a LKB NOVA ultra-microtome (Stockholm, Sweden), stained with a solution of toluidine blue in 0.1 M borate buffer and observed under a light microscope to check the presence of urothelium and detrusor muscle, respectively. Ultra-thin sections (50/60 nm thick) of the selected areas were obtained with the same ultra-microtome by using a diamond knife and stained with an alcoholic solution of uranyl acetate in methanol (50:50) per 12 min. at 45°C, followed by an aqueous solution of concentrated bismuth subnitrate per 10 min. at RT. At least 10–20 ultra-thin sections from all four samples of each animal were examined under a JEOL 1010 electron microscope (Tokyo, Japan) and photographed.

## Results

### Lamina propria

The *lamina propria* consisted of two differently organized layers: an upper one, the sub-urothelial layer, thin and extremely rich in cells; a deeper one, thick and with the features of a typical submucosa. The border between the two layers was undefined.

#### Sub-urothelial layer

**Figure 1:**
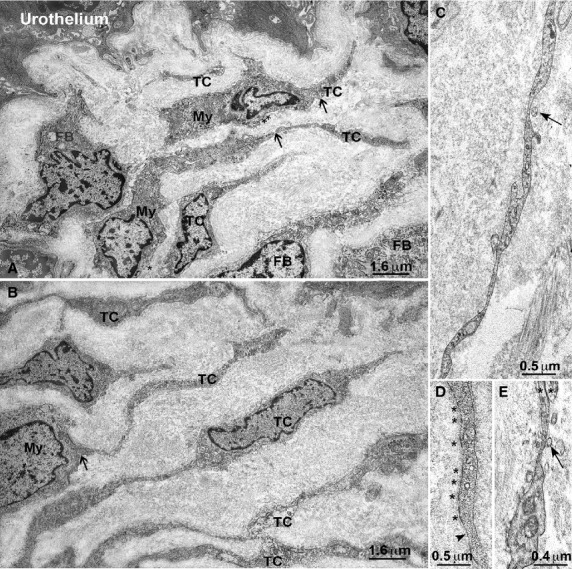
Sub-urothelium. Electron microscopy. (**A**) Several interstitial cell types form layers immediately beneath the urothelium: FB: fibroblasts, TC: telocytes, My: myofibroblasts. *Asterisks* indicate attachment plaques. *Arrows* indicate cell-to-cell contacts between TC and myofibroblasts. (**B**) Many cells with the characteristic features of the telocytes (TC) form layers parallel to the urothelium. The arrow indicate a contact between a TC and a myofibroblast (My). (**C**) Detail of a TC process. The *arrow* indicates a shed vesicle. (**D**) Detail of a TC process. The *arrowhead* indicates a group of caveolae. The *asterisks* indicate several attachment plaques. (**E**) Detail of a TC process. The *arrow* indicates a shed vesicle protruding from the TC process. The two *asterisks* indicate an adherens junction between two TC processes. A and B: bar = 1.6 μm. C and D: bar = 0.5 μm; E: bar = 0.4 μm.

**Figure 2:**
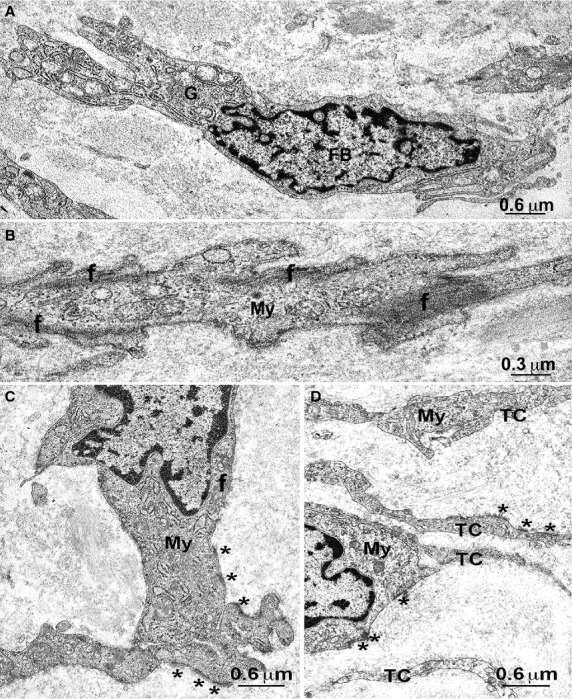
Sub-urothelium. Electron microscopy. (**A**) A fibroblast (FB) with the cytoplasm rich in cisternae of the rough endoplasmic reticulum and with, near the nucleus, a large Golgi apparatus (G). (**B**) Detail of the cytoplasm of a myofibroblast. Bundles of strictly packed myofilaments (f) are located at cell periphery and attached to the plasmalemma. (**C**) A typical myofibroblast (My) with many cisternae of rough endoplasmic reticulum and several attachment plaques (*asterisks*). (**D**) Two myofibroblasts (My) and four telocytes (TC). Both cell types have attachment plaques (*asterisks*). TC and myofibroblats are in contact to each other; the TC in the middle of the figure has an extended contact surface with a myofibroblast. A, C and D: bar = 0.6 μm; B: bar = 0.3 μm.

**Figure 3:**
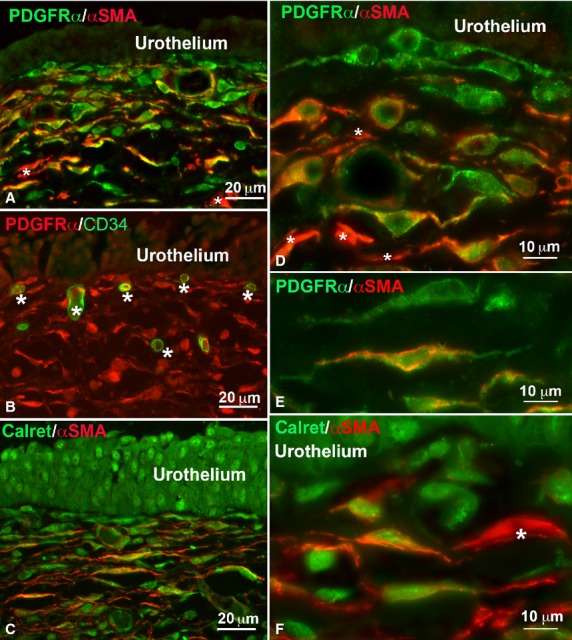
Sub-urothelium. Immunohistochemistry. (**A**) PDGFRα (green) and αSMA (red) immunoreactivity. Immediately beneath the urothelium there are cells identified as telocytes that are PDGFRα-positive only; deeper, these cells are double PDGFRα/αSMA labelled. *Asterisks* indicate cells that, being only αSMA-positive, are identifiable as myofibroblasts. (**B**) PDGFRα (red) and CD34 (green) immunoreactivity. Telocytes are PDGFRα-positive and CD34-negative. The endothelium of blood capillaries is CD34-positive. (**C**) Calret (green) and αSMA (red) immunoreactivity. Urothelial cells and all telocytes (both the αSMA-positive and αSMA-negative ones) are calret-positive. (**D** and **E**) PDGFRα (green) and αSMA (red) immunoreactivity. In **D**, a detail of the two labelling's distribution. Immediately beneath the urothelium, the cells identified as telocytes are PDGFRα-positive only; deeper these cells are double labelled, with the αSMA-positivity mainly distributed in the cell processes and the PDGFRα-positivity in the nucleated portion. *Asterisks* indicate αSMA-positive myofibroblasts. In **E**, a detail of two telocytes one of which, on the upper side, is PDGFRα-positive only and the other, on the lower side, is double labelled. The peripheral labelling of the αSMA is clearly appreciable. (**F**) Calret (green) and αSMA (red) immunoreactivity. Immediately under the urothelium, the telocytes are calret-positive and αSMA-negative; those located deeper are double labelled. Calret-positivity is at the cell periphery and along the cell processes. The *asterisk* indicates a myofibroblast that is αSMA-positive and calret-negative. A–C: bar = 20 μm; D–F: bar = 10 μm.

**Figure 4:**
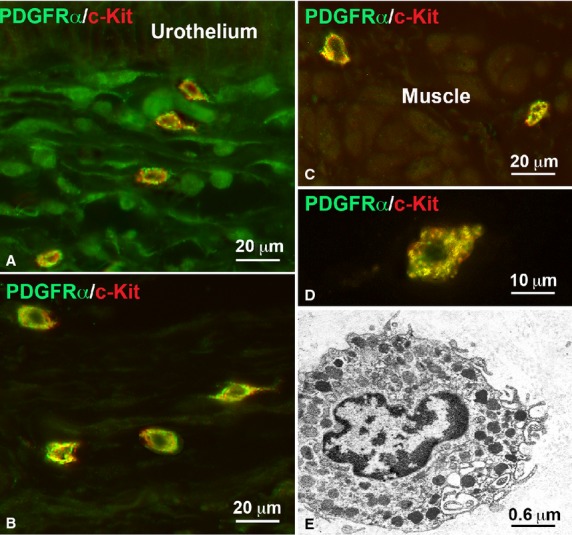
Mast cells. (**A**–**D**) Immunohistochemistry. PDGFRα (green) and c-Kit (red) immunoreactivity. Double labelled cells with a granular cytoplasm are present in the sub-urothelium (**A**), submucosa (**B**) and among the muscle cells of the detrusor (**C**); bar = 20 μm. (**D**) Detail of the PDGFRα/c-Kit double labelling of the mast cell cytoplasm; bar = 10 μm. (**E**) Electron microscopy. A typical mast cell with the cytoplasm filled of granules; bar = 0.6 μm.

**Figure 5:**
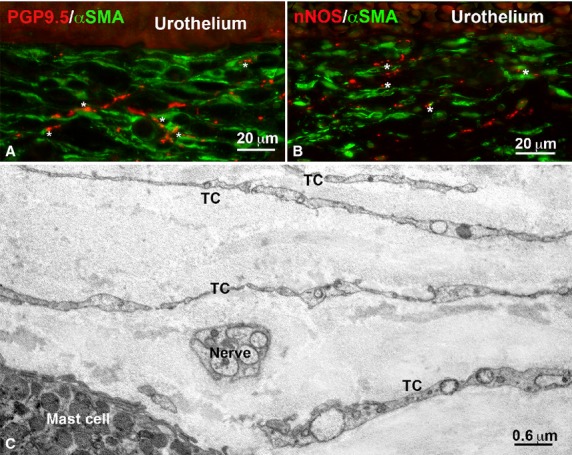
Innervation. (**A** and **B**) Sub-urothelium, immunohistochemistry. (**A**) PGP 9.5 (red) and αSMA (green) immunoreactivity. Varicose nerve fibres are present and many of them (*asterisks*) are in the vicinity of αSMA-positive cells, the presumed TC. (**B**) nNOS (red) and αSMA (green) immunoreactivity. Numerous nNOS-positive nerve endings (*asterisks*) are in the vicinity of the αSMA-positive presumed TC. A: bar = 20 μm; B: bar = 25 μm. (**C**) Electron microscopy: submucosa. Several telocytes (TC) and one mast cell. In the middle a thin nerve bundle; bar = 0.6 μm.

**Figure 6:**
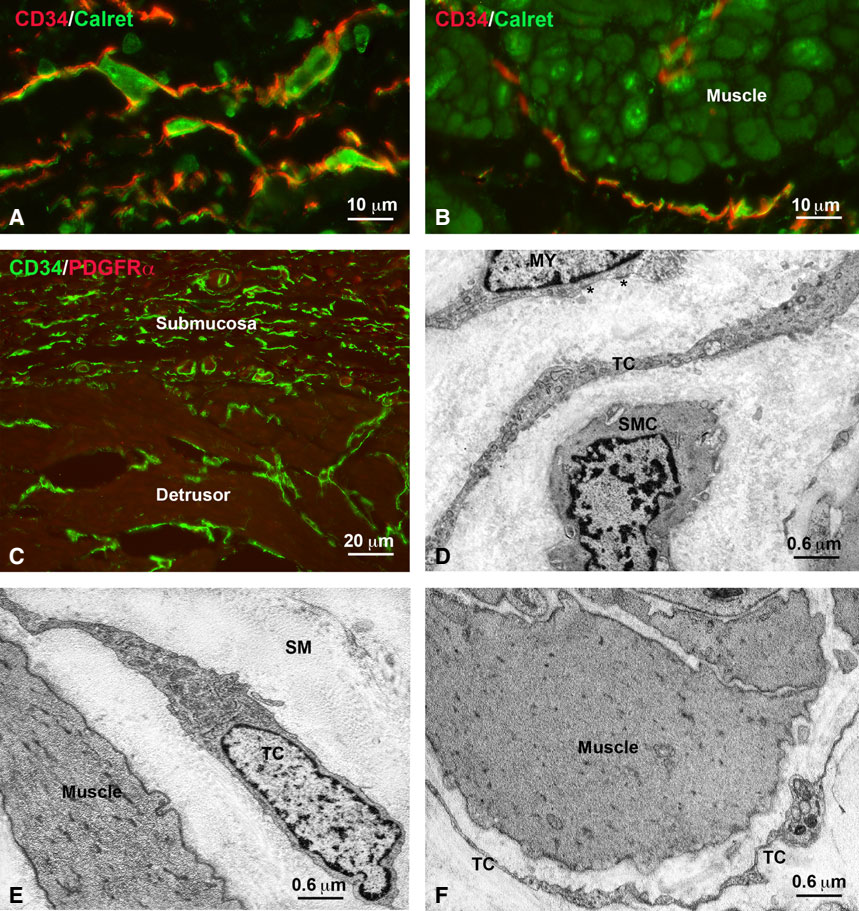
Submucosa and detrusor. (**A** and **B**) Immunohistochemistry: double labelled cells identified as telocytes are CD34 (red) and calret (green) positive in the submucosa and detrusor, respectively. The smooth muscle cells of the detrusor are calret-positive; bar = 10 μm. (**C**) Immunohistochemistry: CD34 (green) and PDGFRα (red) immunoreactivity. Either in the submucosa or the detrusor, the telocytes are CD34-positive and PDGFRα-negative; bar = 20 μm. (**D**–**F**) Electron microscopy. In the submucosa (**D**), at the border between the submucosa and the detrusor (**E**) and around the detrusor muscle bundles (**F**), there are cells with the ultrastructure typical for telocytes; bar = 0.6 μm.

Under TEM, fibroblasts, large oval-shaped cells rich in rough endoplasmic reticulum (RER) and having a large Golgi apparatus (Figs.1A and 2A); myofibroblasts, large oval-shaped cells rich in RER, with a well-developed Golgi apparatus and having bundles of strictly packed myofilaments and extracellular matrix attachments – the fibronexuses (Figs.1A, B and 2B–D); mast cells, oval or fusiform cells with the cytoplasm plenty of electron-dense granules (Figs.4E and 5C) and some immune cells (plasma cells and macrophages, data not shown) were seen. No cell had the features of the ICC, whereas cells with the features reported for the TC formed a thick multilayered area parallel to the urothelial surface. These cells had the characteristic small TC oval body and possessed at least two long, thin, varicose processes (telopodes with alternating podoms and podomeres) (Figs.1A–E, 2D, 5C and 6D–F) through which they contact to each other by unspecialized contacts or junctions adherents (Fig.1E). Shed vesicles close to the telopodes (Fig.1C) or protruding from them (Fig.1E) were frequently seen. Some caveolae were present along the cell processes (Fig.1D). Some of these TC appeared as hybrid cells as they had a larger body containing several RER cisternae, and their cell processes showed attachment plaques with the extracellular matrix similar to the fibronexus typical of the myofibroblasts 24, although smaller and shorter (Figs.1D and 2D) than in these latter cells (Figs.1A, B and 2C, D). Both TC subtypes and myofibroblasts were intermingled and formed a 3D network contacting to each other through epithelial-like but not-specialized cell-to-cell contacts (Figs.1A and 2D). Immunohistochemistry showed that PDGFRα-positive cells, likely identifiable as TC for their shape and arrangement, were present in the entire thickness of the sub-urothelial layer. Those located immediately beneath the urothelium were αSMA-negative (Fig.3A, D, E), while those located deeper and having a larger body were αSMA-positive (Fig.3A, D, E). αSMA-positivity was distributed along the periphery of the cell body and in the processes (Figs.5A, B and 3D–F). Moreover, calret-positivity was detected within the urothelial cells and in all the sub-urothelial TC, either the αSMA-positive or αSMA-negative ones (Fig.3C and F). The cells identifiable as myofibroblasts were αSMA-positive and PDGFRα-negative (Fig.3A and D) and calret-negative (Fig.3F). These cells were especially located in the deeper portion of the sub-urothelium. Blood vessels, in particular capillaries, were more numerous immediately beneath the urothelium (Fig.3B) and the endothelial cells were the unique cell type showing a CD34-positivity in this area. Mast cells only were c-Kit-positive (Fig.4A). These cells were also PDGFRα-positive (Fig.4A). Nerve bundles were small and several nerve endings were close to the cells identified as TC (Fig.5A and B), but under TEM none of them was seen to form cell-to-cell specialized contacts with these cells (Fig.5C). Especially under the urothelium, many of the nerve endings were nNOS-positive (Fig.5B).

#### Submucosa

Under TEM, typical TC were seen scattered within the submucosa thickness (Fig.6D and E). All of them were CD34/calret-positive, but PDGFRα/αSMA/c-Kit-negative (Fig.6A and C). αSMA-positive but CD34/PDGFRα-negative cells were also present and identifiable either as myofibroblasts, mainly located close to the border with the sub-urothelium, or as smooth muscle cells, that formed small bundles in the middle of the submucosa thickness (data not shown). Blood vessels were numerous and attention has to be paid in not confusing the endothelial cells with the TC, being both of them CD34-positive. Mast cells were c-Kit/PDGFRα-positive (Fig.4B) and under TEM no cell with the features of the ICC could be identified. Nerves were present everywhere, but nerve endings were fewer than in the sub urothelial layer.

### Detrusor

The smooth muscle bundles of the detrusor were surrounded by a typical *stroma* and cells with the characteristic ultrastructural features of the TC were present at its submucosal border (Fig.6C and E) and around the muscle bundles (Fig.6B and F). All these cells were CD34/calret-positive and PDGFRα/c-Kit-negative (Fig.6A–C). Under TEM, no cell had the features of the ICC. As in the *lamina propria,* mast cells were c-Kit/PDGFRα-positive (Fig.4C) and endothelial cells CD34-positive (Fig.6C). Nerve bundles and nerve endings were very numerous (data not shown).

## Discussion

The present findings demonstrated that in the human urinary bladder wall, there were ICs sharing several characteristics typical of the TC 26–32; thus, we considered them as TC. However, both TEM and immunohistochemistry revealed that these cells differed to each other in some ultrastructural features and immunohistochemical labelling according to their location, *i.e*. the sub-urothelial layer or the submucosa and the detrusor, respectively. Therefore, on the basis of these findings, we reasonably conclude that in the human urinary bladder there are at least three TC subtypes, two of which located in the sub-urothelium and one in the submucosa and detrusor.

### Sub-urothelium

Immediately beneath the urothelium there was a TC subtype that, at variance with the typical TC 26, contained several cisternae of RER. This subtype was PDGFRα/calret-positive and αSMA/CD34/c-Kit-negative. Deeper in the sub-urothelium, there were TC similar to the previous one for their ultrastructure and immunolabelling but, similarly to the myofibroblasts, these cells were αSMA-positive and possessed attachment plaques. However, this second TC subtype has not to be confused with the myofibroblasts as the latter cells are PDGFRα/calret-negative, have a large body, numerous RER cisternae, a well-extended Golgi apparatus, myofilament bundles and fibronexuses. Rather, these TC can be considered a hybrid cell type sharing with myofibroblasts few TEM characteristics and the αSMA-positivity. Likely, these TC correspond to cells recently identified at this same location in the human bladder and called ‘fibroblasts with myoid differentiation’ 5. Remarkably, all the sub-urothelial TC and the myofibroblasts established extended regions of cell-to-cell contacts, thus forming a mixed network.

In both the submucosa and detrusor, there was a cell type undoubtedly identifiable as TC (or ICLC) 5,8 for its typical ultrastructure and CD34-positivity. We presently ascertained that this third TC subtype was also calret-positive but c-Kit/PDGFRα/αSMA-negative. Therefore, at variance with the gastrointestinal TC which are CD34/PDGFRα-positive 34, in the submucosa of the human urinary bladder none of the cells identified as TC showed this double positivity. Submucosal myofibroblasts also were clearly identifiable, not only for their ultrastructural features and αSMA-positivity, but also for their PDGFRα/CD34/calret-negativity.

The presence of CD34-negative and αSMA-positive TC in the sub-urothelium of human bladder is in agreement with the report by Gevaert *et al*. 3–5, but we also observed immediately beneath the urothelium a second TC subtype that was αSMA-negative. Similarly to us, in the human bladder detrusor, Rasmussen *et al*. 8 described CD34-positive and c-Kit-negative cells (called at that time ICLC). Similar to us, PDGFRα/αSMA-positive cells were recently observed in the sub-urothelium of human bladder 5, but we found also a second TC population that was PDGFRα-positive/αSMA-negative. Interestingly, while the two subtypes of sub-urothelial TC we found were PDGFRα-positive, none of the submucosal and detrusor TC showed this positivity. Conversely, PDGFRα-positive cells (called FLC or IC) were described in the entire guinea pig, rat and mouse bladder wall 7,11,33 and in the human detrusor 33. Briefly, for their immunolabelling, the two sub-urothelial TC subtypes might be analogue to the cells called FLC/IC/PDGFRα-positive cells seen in several laboratory mammals. To note, we found that in the entire human bladder wall, mast cells only were c-Kit-positive. In the human detrusor, Monaghan *et al*. 33 called IC cells having the same labelling and shape of those we identified as mast cells. Moreover, we found that mast cells were also PDGFRα-positive. Although it is desirable to reach a general agreement in considering TC, FLC and IC as the same cell type, a discrepancy between the present data on the location of the PDGFRα-positive cells and those of literature is evident. There is the possibility, however, that this discrepancy is caused by the different animal species and/or to the technical procedures.

Another important matter of debate is the ICC presence in the urinary bladder wall. ICC presence is based on the observations of c-Kit-positive cells 12–19, but some authors did not found any c-Kit-positive cells 5,7. Presently, by using the same antibody previously used in the human gastrointestinal tract to label the ICC and distinguish them from the PDGFRα/CD34-positive cells, we identified as TC (34), we found c-Kit-positive cells, but all of them were mast cells. Identical results have been reported by Gevaert *et al*. 5. TEM investigations in guinea pig and man showed that the presumed ICC did not share the classical ICC features 8,11,17,18 and the present study confirmed the absence at any level of the human bladder wall of cells having the ICC ultrastructural features. Noteworthy, we found that the urinary bladder TC were also calret-positive, independently on their location. This marker was never tested before for these cells, but it has to be mentioned that it is shared with several other cell types, ICC included (rat colon, personal observation; guinea pig intestine) 36. In a recent review 11, likely in an attempt to avoid confusion with the canonical ICC, the urinary c-Kit-positive cells were called IC. In our opinion, to insist in trying to find a similarity with another apparatus, such as the gut, might induce to erroneous interpretations. It a next future a less undetermined name for this peculiar urinary IC type(s) has to be found. C

In summary, taking into account all the findings available, the presents included, a complex picture on the IC types located in the urinary bladder wall comes out. Nevertheless, we believe that our data could help in making some order in attributing an identity at each IC type; especially to the cells we called TC. Indeed, according to the present data, the ICs located in the human urinary bladder we identified as TC likely correspond to the cells called FLC, PFGFRα-positive and IC. Moreover, on the basis of their ultrastructure and immunolabelling, the urinary TC are neither ICC nor myofibroblasts. Finally, although some TC subtypes are PDGFRα-positive as mast cells, they differ from these latter cells, for their c-Kit-negativity and calret-positivity.

In conclusion, in the human bladder there are three types of TC when looking at their ultrastructure and two types on the basis of their PDGFRα/CD34 immunolabelling. Moreover, the PDGFRα-positive TC are distinct in two subtypes whether they are also αSMA-positive. Therefore, the urinary bladder contains peculiar TC likely adapting their morphology to the organ activity. Considering their shape and that all the TC types form networks, we believe that, in all location, these cells, as in the gut, are involved in building a 3-D scaffold able to follow the bladder wall distension and relaxation and, at the same time, avoiding anomalous wall deformation. The ultrastructural complexity of the TC have brought several authors to hypothesise that these cells might have trophic function or be involved in integrating neural processes 37. In this regard, the TC in the sub-urothelium form a mixed network with the myofibroblasts, and, as do these latter cells, make specialized junctions with the extracellular matrix. Noteworthy, this part of the *lamina propria,* together with the urothelium, is considered a sort of sensory region in mediating most of the afferences regulating the micturition reflex 38 and it has been suggested that the resident cells, the extracellular matrix and the numerous nerve endings herein located might function as an integrated system able to interact during any change in the bladder wall pressure 9,10,38. In this regard, the nerve endings, many of which nNOS-positive, observed in the *lamina propria* in close proximity to the TC reinforce the possibility that these cells might be involved in bladder reflexes.
